# Reduction Kinetic of Water Soluble Metal Salts by *Geobacter sulfurreducens*: Fe^2+^/Hemes Stabilize and Regulate Electron Flux Rates

**DOI:** 10.3389/fmicb.2022.909109

**Published:** 2022-06-17

**Authors:** Maksym Karamash, Michael Stumpe, Jörn Dengjel, Carlos A. Salgueiro, Bernd Giese, Katharina M. Fromm

**Affiliations:** ^1^Department of Chemistry, University of Fribourg, Fribourg, Switzerland; ^2^Department of Biology, University of Fribourg, Fribourg, Switzerland; ^3^Associate Laboratory i4HB – Institute for Health and Bioeconomy, School of Science and Technology, NOVA University Lisbon, Costa da Caparica, Portugal; ^4^UCIBIO – Applied Molecular Biosciences Unit, Chemistry Department, School of Science and Technology, NOVA University Lisbon, Costa da Caparica, Portugal

**Keywords:** reaction kinetic, *c*-cytochrome, *Geobacter sulfurreducens*, remediation, bioelectrochemistry

## Abstract

*Geobacter sulfurreducens* is a widely applied microorganism for the reduction of toxic metal salts, as an electron source for bioelectrochemical devices, and as a reagent for the synthesis of nanoparticles. In order to understand the influence of metal salts, and of electron transporting, multiheme *c*-cytochromes on the electron flux during respiration of *G. sulfurreducens*, the reduction kinetic of Fe^3+^, Co^3+^, V^5+^, Cr^6+^, and Mn^7+^ containing complexes were measured. Starting from the resting phase, each *G. sulfurreducens* cell produced an electron flux of 3.7 × 10^5^ electrons per second during the respiration process. Reduction rates were within ± 30% the same for the 6 different metal salts, and reaction kinetics were of zero order. Decrease of *c*-cytochrome concentrations by downregulation and mutation demonstrated that *c*-cytochromes stabilized respiration rates by variation of their redox states. Increasing Fe^2+^/heme levels increased electron flux rates, and induced respiration flexibility. The kinetic effects parallel electrochemical results of *G. sulfurreducens* biofilms on electrodes, and might help to optimize bioelectrochemical devices.

## Introduction

**FIGURE 1:**
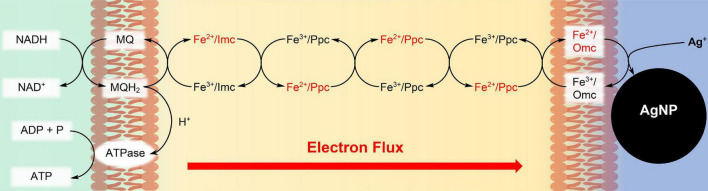
Synthesis of Ag nanoparticles (AgNPs) during respiration of *G. sulfurreducens* with water soluble Ag^+^ ions. The *c*-type cytochromes at the inner cell membrane (Imc), in the periplasm (Ppc), and at the outer cell membrane (Omc) transport electrons from intracellular electron donors like NADH to Ag^+^/Omc complexes, which leads to AgNPs, attached to the outer cell membrane (Chabert et al., 2020). In recent cell growth experiments with CrO_4_^2–^ (Gong et al., 2018), Cr^3+^ reduction products could be detected within the cells, if the bacteria reacted for several hours with the chromium salts. Therefore, our reduction experiments of CrO_4_^2–^ might also occur partly inside of the cells, although the reaction conditions are different.

*Geobacter sulfurreducens* has found important applications in remediation of oxidizing and toxic metal salts (Lovley et al., 2011; Pushkar et al., 2021), as an electron source of microbial fuel cells (Bond and Lovley, 2003; Slate et al., 2019), as well as a reagent for the synthesis of nanoparticles (Lloyd et al., 2011; Khan et al., 2020; Egan-Morriss et al., 2022). This microorganism, first discovered and isolated by Lovley et al. (1987) and Caccavo et al. (1994) relies on different extracellular minerals (Lovley and Phillips, 1988; Shi et al., 2016) and metal salts as electron acceptors (Ding et al., 2008; Lloyd et al., 2011; Lovley et al., 2011; Levar et al., 2014). Respiration experiments on insoluble minerals, and electrochemical studies on solid electrodes have shown that the electron donor NADH in the cytoplasm and the extracellular electron acceptors are separated from each other by the periplasm, delimited, respectively, by the inner and by the outer cell membranes. Electron transport occurs *via* multi-heme-bearing cytochromes (Ueki, 2021), some of which are soluble in the periplasm (Ppc), while others are attached to the inner (Imc) or the outer cell membrane (Omc). Cytochromes of *G. sulfurreducens*, which are involved in the respiration process, exist in many varieties and contain on average 7.5 iron-hemes (Ueki, 2021), and the total number of iron-hemes per cell is about 10^7^ (Esteve-Núñez et al., 2008). Electron transfer through the periplasm is mainly based on triheme cytochromes (Ppc), some of them as protein clusters (Santos et al., 2015). During the stationary phase of the bacteria, all iron/hemes are in the Fe^2+^ state. They become rapidly oxidized to Fe^3+^/hemes (Chabert et al., 2020) upon addition of metal salts with appropriate redox potentials (Santos et al., 2015; Levar et al., 2017). Fe^3+^/hemes then oxidize NADH *via* the menaquinol/menaquinone pool, and the resulting proton gradient catalyzes ATP synthesis. In a recent study on the formation of Ag nanoparticles (AgNPs) by *G. sulfurreducens* respiration with water soluble AgNO_3_, we have demonstrated that Ag^+^ ions are bound by outer membrane cytochromes with high complexation constants (Chabert et al., 2020). Subsequent electron transfer in the Ag^+^/Omc complexes triggered a fast electron flux through *G. sulfurreducens*, leading to AgNPs at the outer cell membrane (Figure 1). The constant electron flux rate of 3⋅10^5^ e^–^⋅s^–1^ per cell was independent of the Ag^+^ ion concentration, and agreed well with electrochemical measurements on single cells of *G. sulfurreducens* (Jiang et al., 2013), as well as *Shewanella oneidensis* (Gross and El-Naggar, 2015). We have now measured reduction rates, and kinetic orders of additional water-soluble metal salts by *G. sulfurreducens*. [Fe(edta)]^–^, [Fe(CN)_6_]^3–^, Co[(bpy)_2_CO_3_]^+^, [VO_2_(edta)]^3–^, CrO_4_^2–^, and MnO_4_^–^ ions were chosen as oxidants, because their concentrations, and the redox changes of *c*-cytochromes could be exactly determined during the fast respiration processes by time resolved experiments. *G. sulfurreducens* cells in the resting and the exponential growth phases were used. Their *c*-cytochrome concentrations were changed by downregulation and mutation.

## Materials and Methods

### Preparation of *Geobacter sulfurreducens* Solutions

*G. sulfurreducens* (DSM-12127) was received from the Leibniz Institute DSMZ. Preparation of standard *G. sulfurreducens* solutions in growth medium A: 5 ml of the purchased bacteria solution were solved in 50 ml of growth medium A (Supplementary Figure 1), which contained in the first growth round 10 mM KCl and 100 μM FeSO_4_. After 5 days of growing, 5 ml of this bacterial solution was added into 50 ml of a growth medium A that contained 2 μM KCI and 25 μM FeSO_4_. This growing procedure with 25 μM FeSO_4_ took about 3–5 days until the fumarate was consumed, and was repeated 4 times. Inductively coupled plasma optical emission spectroscopy (ICP-OES) showed that after this growth process, the bacterial solution of *G. sulfurreducens* contained ≤ 10 μM iron ion concentrations. These standard solutions in medium A without fumarate, which contained *G. sulfurreducens* in the resting state (lag phase) and acetate as carbon source (Estevez-Canales et al., 2015), were directly used for the reduction of the water-soluble metal salts. Preparation of *G. sulfurreducens* solutions in growth medium B: 5 ml of a standard *G. sulfurreducens* solution, which was prepared in growth medium A, and contained 10 μM Fe^2+^, was added to 50 ml of growth medium B lacking FeSO_4_ (Estevez-Canales et al., 2015; Supplementary Figure 1). After 5 days, these solutions in growth medium B, which contained *G. sulfurreducens* in the resting state and acetate as a carbon source (Estevez-Canales et al., 2015), were directly used for the reduction of the water soluble metal salts. The decrease of *c*-cytochrome amounts in *G. sulfurreducens* by growth in medium B compared to medium A was determined by mass spectrometric proteome analysis: disruption of cells and protein extraction were done in a sample homogenizer after adding a lysis buffer (8 M urea, 50 mM Tris-Cl, pH 8) and glass beads (0.18 mm). The same protein amount for each sample was further processed as described in Stekovic et al. (2020). MS raw files were analyzed using the Spectronaut software version 15.7 (Bruderer et al., 2015) with standard settings (without data imputation) in direct DIA mode using reference proteome of *G. sulfurreducens* (UniProt, UP000000577) and common contaminants. Further data processing and statistical analysis used the Perseus software version 1.6. The results are shown in Supplementary Figure 2 and Table 1. The mutant lacking OmcBEST of *G. sulfurreducens* (PCA) was provided by Derek R. Lovley (University of Massachusetts, Amherst, United States). One ml was cultured in 10 ml of NBAF medium under anaerobic conditions as described in Coppi et al. (2001). After 5 days of growing, when *G. sulfurreducens* was again in the lag phase, 5 ml were solved in 50 ml of growth medium A, and reacted for 5 days until *G. sulfurreducens* was again in the lag phase. This growing procedure was repeated 4 times, and the solutions were used directly for the kinetic experiments with water-soluble metal salts.

**TABLE 1:** Functions and remaining percentages of *c*-cytochromes that were downregulated by at least 50% during fumarate-respiring growth of *G. sulfurreducens* in medium B compared to growth in medium A (100%).

*c*-cytochrome	Medium B, %	EET	Predicted cellular location
PpcE (GSU1760)	50	Only found in cultures with Fe^3+^ citrate vs. Fe^3+^ oxides (Ding et al., 2008)	Periplasm
PpcF (GSU2201)	47	Upregulated in cells grown on Fe^3+^ and Mn^4+^ oxide compared to Fe^3+^ citrate (Aklujkar et al., 2013)	Periplasm
GSU3332	47	Gene knockout deficient in the reduction of U^6+^ and Fe^3+^ hydroxide (Shelobolina et al., 2007)	IM (Predicted by Loctree)
CccA (GSU2811)	43	Upregulated by growth on Fe^3+^ and Mn^4+^ oxide compared to Fe^3+^ citrate (Aklujkar et al., 2013)	Periplasm
CoxB (GSU0222)	43	Upregulated by growth on Mn^4+^ oxide compared to Fe^3+^ citrate (Aklujkar et al., 2013)	Periplasm
GSU1740	43	Upregulated by growth on Fe^3+^ and Mn^4+^ oxide compared to Fe^3+^ citrate (Aklujkar et al., 2013)	Periplasm
GSU2210	42		
CcpA (GSU2813)	42		
OmcI (GSU1228)	42	Deletion mutant affected growth in Fe^3+^ citrate and on Fe oxides (Aklujkar et al., 2013)	Periplasm, OM-bounded (Predicted by Loctree)
OmcX (GSU0670)	42	Required for Fe^3+^ reduction (Butler et al., 2010). Downregulated in Fe oxides (Kato et al., 2013)	
OmcA (GSU2884)	40	Upregulated by growth on Fe^3+^ oxides (Aklujkar et al., 2013)	
GSU2743	38	Not involved in EET (Embree et al., 2014)	Periplasm
ppcA (GSU0612)	38	Upregulated by growth on Mn^4+^ oxide (Aklujkar et al., 2013)	Periplasm
ExtG (GSU2724)	33	ExtEFG deletion mutant presented lower levels of Fe^3+^ citrate reduction (Otero et al., 2018)	OM complex ExtEFG
MacA (GSU0466)	29	Upregulated on Mn oxides. Knockout mutant: slow growth on Fe citrate or oxide (Aklujkar et al., 2013)	IM/Periplasm
OmaC (GSU2732)	28	Essential for iron reduction together with OmabcB (Otero et al., 2018)	OM complex OmabcC
PpcD (GSU1024)	25	Upregulated by growth on Fe^3+^ oxide compared to Fe^3+^ citrate (Ding et al., 2008)	Periplasm
PccJ (GSU2494)	23	Upregulated by growth on Fe^3+^ oxides. Mutant had phenotype as wild type (Aklujkar et al., 2013)	Periplasm

*The annotation number for each c-cytochrome encoding gene is given in parenthesis. IM is the abbreviation for inner membrane, and OM for outer membrane.*

### Analysis of Oxidizing Metal Salts

**FIGURE 2:**
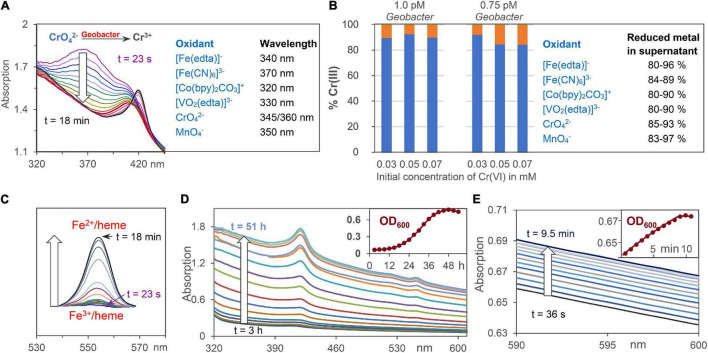
Analytical tools for the analysis of extracellular metal ion, Fe^2+^/heme, and cell concentrations. **(A)** Reduction of oxidizing metals salts by *G. sulfurreducens* by time dependent UV/Vis spectroscopy at wavelengths where product absorptions are negligible. CrO_4_^2–^ respiration is shown as an example. **(B)** ICP analysis of Cr^3+^ ions outside of the cell (blue) and at the cell membrane (orange) after reduction of CrO_4_^2–^. Percentage data for reduced metal salts in the supernatant are given. **(C)** Changes of Fe^2+^/heme concentration during respiration using the areas of Q-bands. **(D)** Increase of UV/Vis absorptions during bacteria growth with fumarate as intracellular oxidant. The insert shows scattering increases at 600 nm (OD_600_) during 2 days of cell growth. **(E)** UV/Vis absorptions during cell growth using low chromate concentrations for *G. sulfurreducens* respiration, starting from the resting state.

Na[Fe(edta)], K_3_[Fe(CN)_6_], K_2_CrO_4_, and KMnO_4_ were purchased from Sigma-Aldrich. Na_3_[VO_2_(edta)] was synthesized according to Komarova et al. (1991). [Co(bpy)_2_CO_3_]Cl was generated from [Co(bpy)_3_]Cl_3_, which was solved in growth medium A without fumarate and acetate. O_2_ was exchanged by N_2_/CO_2_ (80/20) and the solution was heated to 125°C for 20 min at 1.25 bar. The structure of [Co(bpy)_2_CO_3_]Cl was confirmed by single crystal X-ray diffraction (SC-XRD) and electrospray ionization mass spectrometry (ESI-MS). Concentration decrease of the Fe^3+^, Co^3+^, V^5+^, Cr^6+^, and Mn^7+^ salts, which were reduced by *G. sulfurreducens* to Fe^2+^, Co^2+^, V^4+^, Cr^3+^, and Mn^4+^ salts, respectively, was analyzed by UV/Vis spectroscopy at wavelengths shown in Figure 2A and Supplementary Figure 3. In order to determine the location of the oxidizing metal salts after *G. sulfurreducens* respiration, solutions of 4.65 mM Na[Fe(edta)], K_3_[Fe(CN)_6_], [Co(bpy)_2_CO_3_]Cl, Na_3_[VO_2_(edta)], as well as 1.55 mM K_2_CrO_4_ and KMnO_4_, respectively, were treated for 20 min with N_2_/CO_2_ (80/20) at ambient temperature. Then, 0.2 ml of them were added to 6 ml of a standard *G. sulfurreducens* solution. After 1 h reaction time (30°C) 0.35 ml of a 37% HCl solution were added, mixed with a vortex for about 1 min, and centrifuged at 10,000 rpm at 20°C for 10 min. Inductively coupled plasma optical emission spectroscopy (ICP-OES) demonstrated that about 90% of the metal salts were observed outside of the cells, and analysis of the Cr^3+^ distribution (Gong et al., 2018) showed that up to 93% of the chromium ions were found in the supernatant, about 6% at the cell membrane, and less than 1% was detected inside of the cells (Figure 2B).

### Analysis of Fe^2+^/Hemes

Concentrations of Fe^2+^/hemes were analyzed by their Q-band areas between 540 and 570 nm (Figure 2C), and in some cases also by their Soret band at 420 nm (Figure 2A). Ultrasound treatment, which destroyed the cell membranes of *G. sulfurreducens*, did not increase the total UV/Vis absorption of iron-hemes. This demonstrates that *G. sulfurreducens* cells are transparent enough to detect all iron-hemes of the bacteria. Filtration of *G. sulfurreducens* solutions gave a tiny peak (≤5%) of iron hemes in the supernatant (Supplementary Figure 4). With about 10^7^ iron hemes per cell (Esteve-Núñez et al., 2008) and 10^–12^ M cell concentrations of our experiments, these 5% lead to less than 1 μM Fe^2+^/heme solutions outside of the cell, which could at best reduce less than 1% of 0.1 mM extracellular metal ion salt solutions.

### Analysis of Cell Growth

Cell growth was analyzed by spectroscopy at 600 nm (Muhamadali et al., 2015). *G. sulfurreducens* cells are about 1–2 μm large, thus a concentration increase raised the light scattering effect on the UV/Vis spectra (Figure 2D). Test experiments with 40 mM fumarate as an internal oxidant for the cell growth proved that OD_600_ data followed the same exponential increase as experiments, where cell growth in solution was determined by increase of the cell weight (Engel et al., 2020). Dilution of clear cell solutions changed the OD_600_ values in a linear way, and an OD_600_ value of 0.54 corresponds to 0.7 pM *G. sulfurreducens* (Vasylevskyi et al., 2017). The change of OD_600_ values during respiration was detected with high accuracy, so that it could also be used to follow cell growth in experiments with low oxidant concentrations (Figure 2E). Rates of metal salt induced cell growth were measured at 30°C under anaerobic conditions: 0.1 ml of a K_3_[Fe(CN)_6_] solution was added to 3 ml of a standard *G. sulfurreducens* solution. The initial concentration of the oxidant in the reaction mixture was 0.15 mM, and the cell growth was analyzed at OD_600_. Analogous experiments with K_2_CrO_4_ were carried out with reaction mixture concentrations of 0.03 and 0.05 mM.

### Kinetic Experiments of Metal Salt Reduction by *Geobacter sulfurreducens* Starting From the Resting (Lag) Phase

**FIGURE 3:**
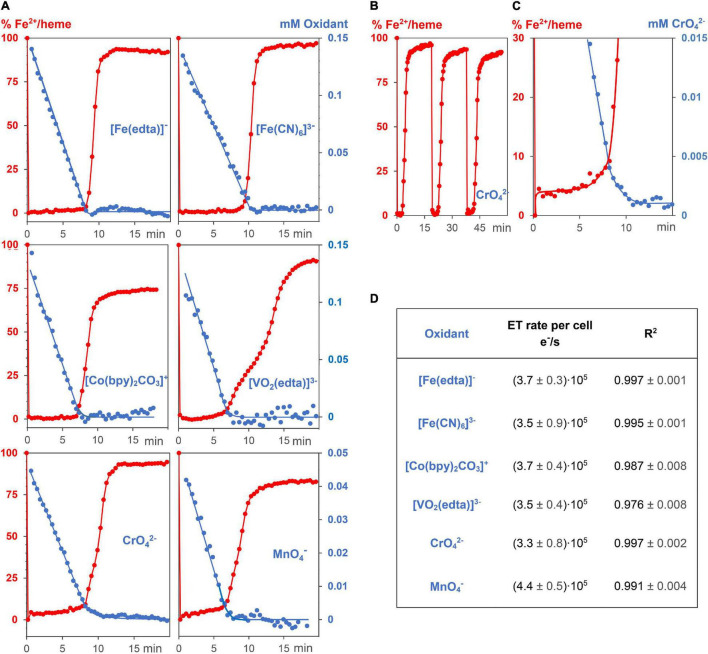
Kinetic experiments of *G. sulfurreducens* with extracellular metal salts. **(A)** Reduction of 6 different oxidizing metal salt solutions (blue, 0.05–0.15 mM) with *G. sulfurreducens* solutions in the resting state (8.9 ± 0.3 pM). Their linear time dependences of the metal salt concentrations demonstrate zero kinetic order of the reduction process. At the start of the experiments, all iron ions of the bacterial multiheme cytochromes were in the Fe^2+^ state (red). They were rapidly oxidized upon addition of extracellular metal salts, their levels remained low during reduction of the metal salts, and increased again after major consumption of the oxidants. **(B)** Experiments could be repeated several times. Shown are Fe^2+^/heme levels upon three consecutive additions of CrO_4_^2–^. **(C)** A closer look at the Fe^2+^/heme (red) and CrO_4_^2–^ (blue) concentrations. The Fe^2+^ /heme levels increased after major part of CrO_4_^2–^ was reduced, which obviously stabilized EET rates at low concentrations of the extracellular metal salts. **(D)** Zero order reduction rates per cell for the 6 different metal salts at 30°C and pH = 7.4.

Kinetic measurements were carried out in standard *G. sulfurreducens* solutions in the lag phase (see above) with 0.15 mM Na[Fe(edta)], K_3_[Fe(CN)_6_], Co[(bpy)_2_CO_3_]Cl, Na_3_[VO_2_(edta)], and 0.05 mM K_2_CrO_4_ and KMnO_4_ solutions, respectively. Under these conditions, the oxidizing metal salts did not kill the bacterial cells, and the reaction mixtures remained homogeneous. All experiments were repeated 3 times at 30°C under N_2_/CO_2_ (80/20). Addition of 0.05 or 0.1 ml metal salt solutions to 3 ml standard *G. sulfurreducens* in the lag phase occurred by injection through a sealing plug with needles that had been sterilized with a Bunsen burner. Oxidants were used in such amounts that their initial concentrations in the reaction mixtures were 0.05 mM for Cr^6+^ or Mn^7+^, and 0.15 mM for Fe^3+^, Co^3+^, and V^5+^, respectively. The concentrations of *G. sulfurreducens* were calculated from the OD_600_ data. UV/Vis spectra were recorded between 610 and 320 nm. Each run took 25.3 s. Concentration changes of metal salts were analyzed at wavelengths that are listed in Figure 2A and Supplementary Figure 3. Concentration changes of Fe^2+^/hemes were determined by the areas of the Q-bands (540, 570 nm) if Na[Fe(edta)], K_3_[Fe(CN)_6_], KMnO_4_, and K_2_CrO_4_ were used as oxidants (Figure 2C). Because Co^2+^ and V^4+^ salts absorb at the Q-bands wavelengths, the differences of Soret bands at 420 nm (Fe^2+^/heme) and 410 nm (Fe^3+^/heme) were used. The determined concentrations of oxidizing metal salts and Fe^2+^/hemes were plotted against reaction times. The linear time dependences of the metal salt concentrations (Figure 3A) are the electron flux rates, and division by *G. sulfurreducens* concentrations led to the electron flux rate per cell (Figure 3D).

### Kinetic Experiments of Metal Salt Reduction by *Geobacter sulfurreducens* Starting From the Growth (Log) Phase

**FIGURE 4:**
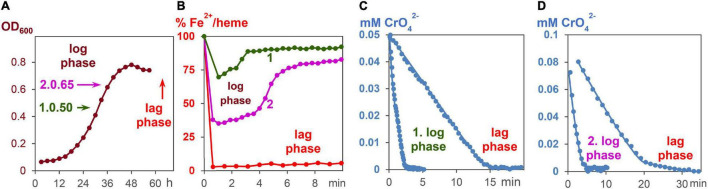
CrO_4_^2–^ induce respiration of *G. sulfurreducens*, starting from the log phase. **(A)** Fumarate- respiring growth of *G. sulfurreducens*. Bacteria after 30 and 36 h growth, respectively, were used for metal induced respiration of *G. sulfurreducens* in the log phase. **(B)** Fe^2+^/heme levels during CrO_4_^2–^ induced respiration of bacteria starting from log phases 1 and 2, as well as the resting (lag) phase. **(C)** Reduction of CrO_4_^2–^ (0.05 mM) by bacteria (0.63 ± 0.2 pM) in the log phase 1 and the lag phase. **(D)** Reduction of CrO_4_^2–^ (0.1 mM) by bacteria (0.83 ± 0.2 pM) in the log phase 2 and the lag phase.

All experiments were carried out under strictly anaerobic conditions at 30°C. To start the growth process with fumarate as an oxidant, 5 ml of the standard *G. sulfurreducens* solution were added to 50 ml of growth medium A. The growing process was analyzed by taking probes every 3 h and measuring the OD_600_ values (Figure 4A). After about 30 h, 3 ml of a *G. sulfurreducens* solution, which was then in the exponential growth (log) phase, was injected with a sterilized needle through a sealing plug into an UV cuvette. The OD_600_ values of *G. sulfurreducens* were about 0.50. To this *G. sulfurreducens* solution, which contained all iron-hemes in the Fe^2+^ oxidation state, 0.05 ml of a K_2_CrO_4_ solution was added, so that the initial concentration of CrO_4_^2–^ was 0.05 mM (experiment 1, Figures 4B,C). Another experiment with *G. sulfurreducens* (OD_600_ = 0.65), which had continued its growth with fumarate as oxidant, was carried out about 6 h later: 3 ml of *G. sulfurreducens* in the exponential growth (log) phase were injected into an UV cuvette, and 0.1 ml of a CrO_4_^2–^ solution was added, so that the reaction mixture was at the start 0.1 mM in CrO_4_^2–^ (experiment 2, Figures 4B,D). Concentration changes of CrO_4_^2–^ and Fe^2+^/hemes were analyzed from the UV/Vis spectra at 345 nm and the Q-band, respectively.

## Results

### Influence of Extracellular Metal Salts on Reduction Rates

Reactions of 0.15 mM Na[Fe(edta)], K_3_[Fe(CN)_6_], Co[(bpy)_2_CO_3_]Cl, Na_3_[VO_2_(edta)], and 0.05 mM K_2_CrO_4_ as well as KMnO_4_ solutions with 0.89 pM *G. sulfurreducens* in the resting phase, solved in media as described above, oxidized the Fe^2+^/hemes of cytochromes to Fe^3+^/hemes within a few seconds, and a steady metal salt reduction occurred over 10 min (Figure 3A). The concentration of the electron transporting Fe^2+^/hemes remained nearly constant at a low level until up to 80% of the metal salt reductions were completed (Figures 3A,C). After about 7–9 min, when most of metal salts had been reduced, Fe^2+^/hemes were regenerated by cellular processes, and the bacteria are ready for a second round of the metal ion reduction process (Figure 3B). The electron flux did not change although concentrations of metal salts decreased. Their linear time dependences are the overall reduction rates, and division by *G. sulfurreducens* concentrations yielded electron flux rates per single cell. Data for CrO_4_^2–^ and MnO_4_^–^ were furthermore multiplied by the stoichiometric factor of 3, considering a three electron transfer. All six metal salts led to the fast electron flux rate of 3.7⋅10^5^ e^–^⋅s^–1^ per cell with a reproducibility of ± 1.2⋅10^5^ e^–^⋅s^–1^ (Figure 3D). Thus, reduction rates were not only independent of metal salt concentrations, but also of the metal salt types. The variation of bacteria or initial metal salt concentrations by a factor of 2 did not change reduction rates per cell (Supplementary Figure 5). A fivefold increase of extracellular metal salts started to deactivate cells, which led to a slowdown of the reduction, indicated by a curvature of the time dependent metal salt reduction, and a lower regeneration of the Fe^2+^/hemes. Additional experiments showed that reduction of metal salts could neither be detected with dead *G. sulfurreducens* cells nor with the supernatant of living cells, and Cr_2_(SO_4_)_3_ or K_3_[Co(CN)_6_], which cannot oxidize Fe^2+^/hemes of cytochromes, did not drive the respiration.

### Influence of Fe^2+^/Hemes on Reduction Rates

**FIGURE 5:**
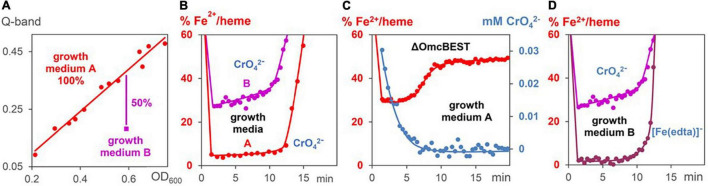
Changes of Fe^2+^/heme levels during metal induced respiration of *G. sulfurreducens*. **(A)** Linear correlation (red line) between Fe^2+^/heme concentrations (Q-bands) and *G. sulfurreducens* concentrations (OD_600_ data). The data (red points) were gained by dilution and different cell growth experiments in medium A. Measurement (magenta point) with *G. sulfurreducens*, grown in medium B, where *c*-cytochromes are downregulated. **(B)** Fe^2+^/heme levels during CrO_4_^2–^ induced respiration (0.05 mM) of cells grown in media A (0.63 pM) and B (0.73 pM). **(C)** Fe^2+^/heme levels (red) and CrO_4_^2–^ concentrations (blue) during CrO_4_^2–^ induced respiration (0.05 mM) of mutants lacking OmcBEST (0.76 pM) grown in medium A. **(D)** Fe^2+^/heme levels of experiments with *G. sulfurreducens* (0.74 pM), grown in medium B, with [Fe(edta)] ^–^ (0.15 mM) and CrO_4_^2–^ (0.05 mM), respectively.

Experiments were carried out with bacteria, which contained either lower *c*-cytochrome concentrations, or reacted faster with the metal salts. A downregulation of *c*-cytochromes was carried out by preparation of *G. sulfurreducens* cells in a growth medium of low Fe^2+^ concentration (medium B). Mass spectrometric proteome analysis detected 2,579 proteins after growth in medium A as well as in medium B, from which 64 are *c*-cytochromes (Supplementary Figure 2). Two thirds of them were downregulated by cell growth in medium B, 18 by more than 50% (Table 1). Comparison with literature data demonstrated that 17 of these 18 cytochromes are involved in the EET process. Most of them are located in the periplasm, from which three are predicted to be bound to the outer membrane, two to the inner membrane, and two cytochromes are outer membrane complexes. As a consequence, the total iron heme concentrations decreased by 50% in the downregulated cells (Figure 5A). Reduction experiments of these bacteria with metal salts showed that electron flux rates remained constant, whereas the Fe^2+^/heme levels increased considerably (Figure 5B). Obviously, constant rates could be maintained in cells of lower *c*-cytochrome concentrations by rising the Fe^2+^/heme levels. A similar effect was observed with a mutant, where outer membrane cytochromes OmcB, OmcE, OmcS, and OmcT were deleted (Figure 5C).

An increase of electron flux rates was achieved by experiments starting with *G. sulfurreducens* in the exponential growth phase (log phase). In order to carry out these measurements, cells were prepared under fumarate-respiring conditions (Butler et al., 2006). The log phase started after several hours, reached a rate maximum at about 30 h, then slowed down, and stopped during the third day (Figure 4A). In the first hours of this growth process, Fe^2+^/hemes were partly oxidized but became reduced again during the log phase (Supplementary Figure 6). Once all iron hemes of *G. sulfurreducens* were in the Fe^2+^ state, K_2_CrO_4_ was added to the growing *G. sulfurreducens* solution, and the CrO_4_^2–^ reduction rate was measured (Figures 4C,D). Experiments at different growth times showed that electron flux was 4–6 times faster in the log phase than with cells starting from the lag phase. Cells in the exponential growth demands faster ATP production, which can be achieved by an increase of the electron flux rate. This was made possible by an increase of the Fe^2+^/heme levels in the *c*-cytochromes from 5% (lag phase) to up to 75% (log phase) during the metal salt reduction (Figure 4B).

### Influence of Extracellular Metal Salts on Cell Growth Rates

**FIGURE 6:**
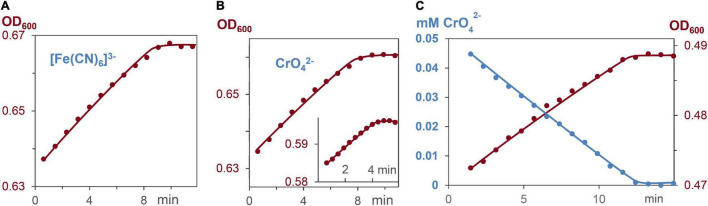
Increase of cell concentration and decrease of oxidants [Fe(CN)_6_]^3–^ and CrO_4_^2–^ during *G. sulfurreducens* respiration in grow medium A, lacking fumarate, and stating from the lag phase. **(A)** Linear increase of the bacterial growth with 0.15 mM [Fe(CN)_6_]^3–^, *R*^2^ = 0.98. **(B)** Linear increase of the bacterial growth with 0.05 and 0.03 mM CrO_4_^2–^ (insert), *R*^2^ = 0.98. **(C)** Linear CrO_4_^2–^ decrease (blue, *R*^2^ = 0.99), and linear bacterial growth (brown, *R*^2^ = 0.98) of the same respiration experiment.

Respiration induces the formation of ATP, which leads to bacterial growth in ATP dependent processes (Brown, 1992; Velten et al., 2007; Bochdansky et al., 2021). To elucidate whether ATP formation and ATP consumption occur with the same rates, the decrease of the metal salts, and the increase of cell growth were measured. Reactions started with *G. sulfurreducens* in the resting state and medium A as solvent, which lacks fumarate as oxidant. Respiration was induced by addition of 0.03–0.05 mM CrO_4_^2–^ or 0.15 mM [Fe(CN)_6_]^3–^ solutions. Figures 6A–C demonstrate that cell growth increased linearly, and required the same reaction times as the respiration process. The OD_600_ increase during respiration revealed that a 0.15 mM electron flux generated 4.5 ± 0.5% cell increase from 0.81 pM *G. sulfurreducens* solutions. Hence, a flow of 0.15 mM electrons produced 0.036 pM cell growth, so that 4⋅10^9^ electrons were needed to synthesize enough ATP for one cell division, which agrees with the analysis of electrochemical experiments (Levar et al., 2013).

## Discussion

Respiration of lag phase *G. sulfurreducens* cells in a growth medium, which lacks the oxidant fumarate, reduced Fe^3+^, Co^3+^, V^5+^, Cr^6+^, and Mn^7+^ ions of the 6 different metal salts with electron flux rates of 3.7⋅10^5^ e^–^⋅s^–1^ per cell at 30°C, a pH of 7.4 and a reproducibility of ± 30% (Figures 3A,D). The nearly constant Fe^2+^/heme levels demonstrated that the oxidation of Fe^2+^/hemes by the metal salts, and the reduction of Fe^3+^/hemes by the menaquinole pool occurred with the same rates, leading to a constant electron flux. Rates and kinetic orders agree well with our earlier reduction experiments of Ag^+^ ions by *G. sulfurreducens* (Chabert et al., 2020), which led to Ag nanoparticles (AgNPs), as reaction products (Figure 1). We had measured high binding constants of Ag^+^ ions to Met and His of outer membrane cytochromes, and observed the formation of AgNPs at the outer cell membrane. In recent cell growth experiments with CrO_4_^2–^ (Gong et al., 2018), Cr^3+^ reduction products could also be detected within the cells, if the bacteria were treated for several hours with metal salts. Our experiments were finished within 10–20 min, and we never observed Cr^3+^ within the cells (Figure 2B), but to avoid overinterpretation, we don’t exclude the possibility that some of the CrO_4_^2–^ was reduced inside of the cells even under our different reaction conditions. This obviously did not change the electron flux rate (Figure 3A), which is driven by the need for a constant ATP production (Velten et al., 2007; Bochdansky et al., 2021; Wilson and Matschinsky, 2021) during *G. sulfurreducens* respiration (Figures 6A–C). Interestingly, the same electron flux rates were also measured in electrochemical experiments on single cells of *G. sulfurreducens* (Jiang et al., 2013) as well as *Shewanella oneidensis* (Gross and El-Naggar, 2015).

The influence of Fe^2+^/hemes on electron flux rates was studied (a) with *G. sulfurreducens* cells of downregulated *c*-cytochromes, (b) with cells in the exponential growth phase, and (c) with *G*. *sulfurreducens* mutants. Decrease of *c*-cytochrome concentrations (Figure 5A and Table 1) was observed in microorganisms that were prepared under fumarate-respiring conditions at very low FeSO_4_ concentrations (medium B). Addition of CrO_4_^2–^ ions to these downregulated cells induced nearly the same reaction rates as experiments with *G. sulfurreducens*, grown in medium A (Figure 5B). This was surprising, as *c*-cytochromes are the electron transporting carriers and their decrease should slow down the electron flux. Obviously, the observed rise of Fe^2+^/heme levels from ≤ 5 to 30% compensated the downregulation of *c*-cytochromes. Electron flux rates of metal salt induced respiration, which used *G. sulfurreducens* cells in the exponential growth phase, were 4–6 times faster compared to experiments starting from *G. sulfurreducens* in the resting phase (Figures 4C,D). Such an acceleration is reasonable as the ATP demand increases in the exponential growth phase, requiring at the same time a faster electron flux, which was achieved by an increase of Fe^2+^:/heme levels in the cytochromes from 5% *via* 40 to 75% (Figure 4B). Such a rise of the Fe^2+^/heme level augments the reductive power of multiheme cytochromes (Quian et al., 2011; Liu and Bond, 2012; Santos et al., 2015), and increases electron transfer rates by the Marcus theory (Marcus, 1993). It demonstrates the importance of the electron storing capacities of multiheme cytochromes (Esteve-Núñez et al., 2008), which can regulate respiration by their redox states. Our observations are again in accord with electrochemical experiments, where the redox status of *c*-cytochromes in *G. sulfurreducens* biofilms changed with the applied potential (Liu et al., 2011). Thus, the Fe^2+^/heme level is an important parameter for the optimization of *G. sulfurreducens* as an electron-producing source.

The analogous effects of our metal salt induced electron flux measurements with electrochemical current production of biofilms at the anode inititated us to measure the reduction rate of CrO_4_^2–^ by *G. sulfurreducens* mutants, in which OmcBEST was deleted. Electrochemical measurements on biofilms had shown (Nevin et al., 2009) that “deletion of OmcS, OmcB and OmcE had nearly no impact on maximum current production.” This is in strong contrast to Fe^3+^ oxide and Fe^3+^ citrate induced cell growth experiments. *G. sulfurreducens* mutants, in which OmcB, OmcE, or OmcS were mutated out (Leang et al., 2003; Richter et al., 2011) reduced the cell growth rates dramatically. Our rate measurements with water-soluble CrO_4_^2–^ ions are again in accord with electrochemical results. Figure 5C demonstrates that the mutant, in which OmcBEST was deleted, hardly changed the CrO_4_^2–^ reduction time, but the missing outer membrane cytochromes induced a drastic increase of the Fe^2+^/hemes level during the metal salt reduction. It demonstrates that the effect of decreasing outer membrane cytochromes on the respiration rate was compensated by an increase of the Fe^2+^/heme level. Thus, Fe^2+^/hemes play a central role in the regulation of electron flux rates. These measurements stimulate studies to elucidate the effects of *G. sulfurreducens* mutations on electron flux rates in bioelectrochemical measurements compared to cell growth experiments. An obvious difference between these two techniques is that in cell growth experiments the cells are several hours in contact to the oxidizing minerals and the reduced metal ions. In contrast, electrochemical experiments with *G. sulfurreducens* biofilms on electrodes, as well as the metal salt induced electron flux measurements, presented in this publication, take only some minutes. We will check in future work, whether the sensitivity of mutated cells against strong oxidants is one of the reasons for the differences between long time cell growth and short time bioelectrochemical experiments.

## Data Availability Statement

The datasets presented in this study can be found in online repositories. The names of the repository/repositories and accession number(s) can be found below: ProteomeXchange, PXD032892.

## Author Contributions

MK designed and carried out the kinetic experiments. MK and BG analyzed the data and calculated reaction kinetics. MS carried out the proteomic experiments. MS, JD, and CS discussed the proteomic experiments. MK, BG, and KF discussed all data and suggested the reaction mechanism. All authors contributed to the article and approved the submitted version.

## Conflict of Interest

The authors declare that the research was conducted in the absence of any commercial or financial relationships that could be construed as a potential conflict of interest.

## Publisher’s Note

All claims expressed in this article are solely those of the authors and do not necessarily represent those of their affiliated organizations, or those of the publisher, the editors and the reviewers. Any product that may be evaluated in this article, or claim that may be made by its manufacturer, is not guaranteed or endorsed by the publisher.
