# Supplementary material for: Reduction Kinetic of Water Soluble Metal Salts by Geobacter sulfurreducens: Fe2+/Hemes Stabilize and Regulate Electron Flux Rates
Source: Front Microbiol. 2022 Jun 17;13:909109. doi: 10.3389/fmicb.2022.909109 (PMC9248073; doi:10.3389/fmicb.2022.909109)
Supplement: Supplementary file 1 [file Data_Sheet_1.PDF]

Supplementary Material

Fig. S1. ***G. sulfurreducens* growth media.**

Compound	**Medium A** Concentration	**Medium B** Concentration
Disodium fumarate	40 mM	40 mM
$NaHCO_3$	29.8 mM	29.8 mM
Na_2CO_3	-	-
$NaCH_3COO$	19.5 mM	20 mM
NH_4CH_3COO	9.3 mM	-
NaH_2PO_4	3.0 mM	0.43 mM
KH_2PO_4	-	-
K_2HPO_4	-	-
KNO_3	1.3 mM	-
L-Cysteine	-	-
Na_2SeO_4	-	-
2,2'-bipyridine	-	2.73 µM
$MgSO_4$	122 µM	410 µM
Nitrilotriacetic acid	78 µM	112 µM
$MnSO_4$	30 µM	-
$MnCl_2$	-	5.053 µM
$FeSO_4$	25 µM	-
$ZnSO_4$	17 µM	7 µM
$CaSO_4$	7 µM	-
$CaCl_2$	-	270 µM
$CoSO_4$	4 µM	-
$CoCl_2$	-	7.15 µM
KCl	2 µM	1.34 mM
NaCl	-	-
NH_4Cl	-	4.7 mM
H_3BO_3	1.6 µM	0.8 µM
Na_2MoO_4	1 µM	3.72 µM
$Ni(NO_3)_2$	1 µM	-
$NiSO_4$	-	4.2 µM
Na_2WO_4	0.75 µM	6.1 µM
$CuSO_4$	0.4 µM	-
$CuCl_2$	-	1.75 µM
Pyridoxine	0.6 µM	0.5 µM
Nicotinic acid	0.4 µM	0.4 µM
p-aminobenzoic acid	0.36 µM	0.36 µM
Lipolic acid	0.24 µM	0.24 µM
DL-Ca pantothenate	0.23 µM	0.21 µM
$AlK(SO_4)_2$	0.2 µM	0.1 µM
Thiamine • HCl	0.2 µM	0.15 µM
Riboflavin	0.13 µM	0.13 µM
Biotin	82 nM	82 nM
Folic acid	45 nM	45 nM
Cyanocobalamin	0.75 nM	0.75 nM

All media were dispensed into anaerobic pressure bottles with butyl rubber stoppers, and bubbled with an 80% N_2–20% CO_2 gas mixture to remove oxygen. Then, they were autoclaved at 125°C and 1.25 bar for 20 min to get them sterilized.

Fig. S2. **Regulation of *c*-cytochromes**

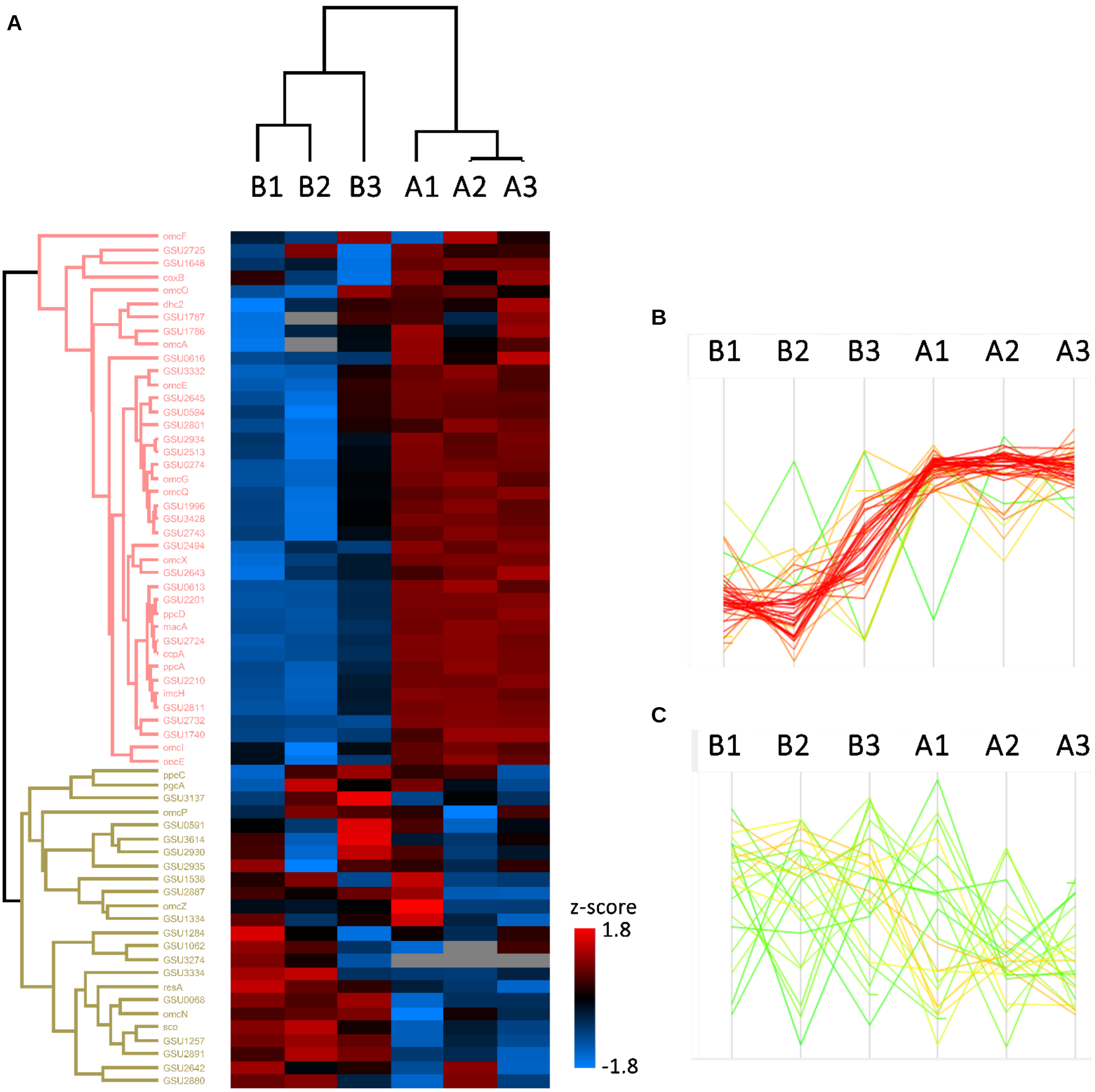

G. sulfurreducens cells were prepared by fumarate-respired growth in media A and B. **(A)** Heatmap: shown are the z-score normalized quantities of identified *c*-cytochromes in media A and B. **(B)** Profile plot of *c*-cytochromes that were downregulated in medium B. **(C)** Profile plot of *c*-cytochromes, where growth medium B had in most cases no unambiguous impact on the amount of *c*-cytochromes, some are slightly upregulated in samples of growth media B.

Fig. S3. **Wavelengths used for the analysis of water soluble metal salt concentrations during kinetic experiments.**

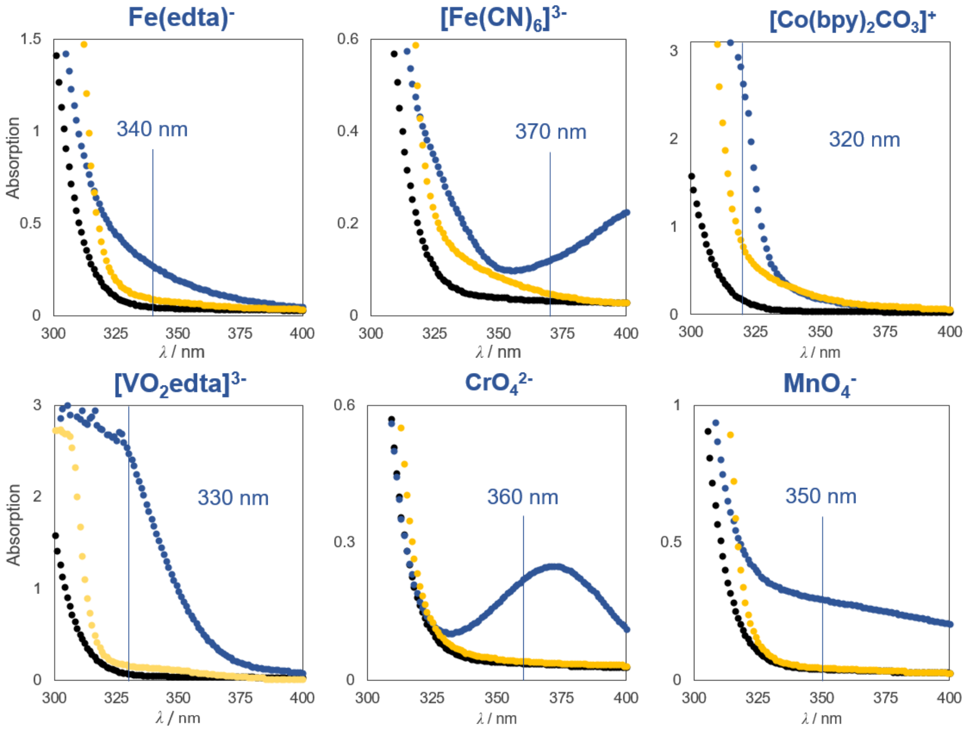

Black lines are absorption spectra of growth medium A without fumarate and acetate. Blue lines are spectra of 0.15mM Na[Fe(edta)], $K_3[Fe(CN)_6]$, $[Co(bpy)_2CO_3]Cl$, and $Na_3[VO_2(edta)]$, as well as 0.05mM $KMnO_4$, and K_2CrO_4, respectively, solved in growth medium A without fumarate and acetate. Yellow lines are the metal salt solutions of oxidants, which were reduced by L-ascorbic acid. The absorption of the reduced Co^{2+} salt is not negligible at the analytical wavelength. This was taken into account in the kinetic experiments.

Fig. S4. **Remaining iron hemes in the supernatant after filtration of the bacteria.**

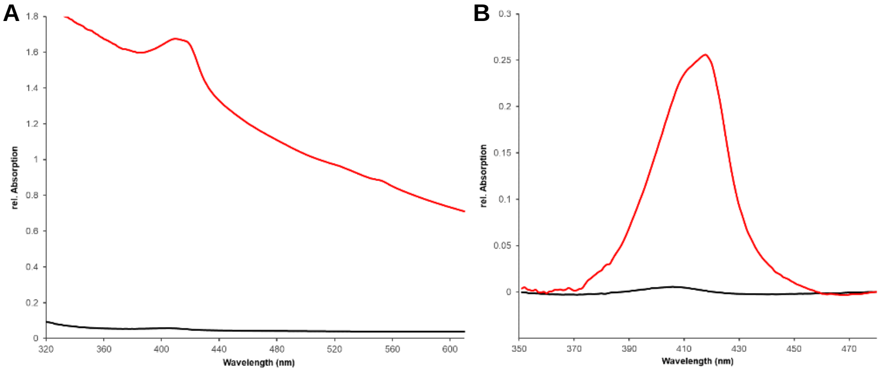

A UV-Vis spectrum of a *G. sulfurreducens* solution in growth medium A without fumarate (red line). UV-Vis spectrum of the supernatant (black line) that was obtained after filtration with hydrophilic PTFE syringe filters (pore size – 0.20 μm). **B** The area of the tiny Soret band in the supernatant (black line) is ≤ 5% of the area of bacterial Soret-band before filtration (red line).

Fig. S5. **Influence of CrO_4^{2-} and *G. sulfurreducens* concentrations on reaction times.**

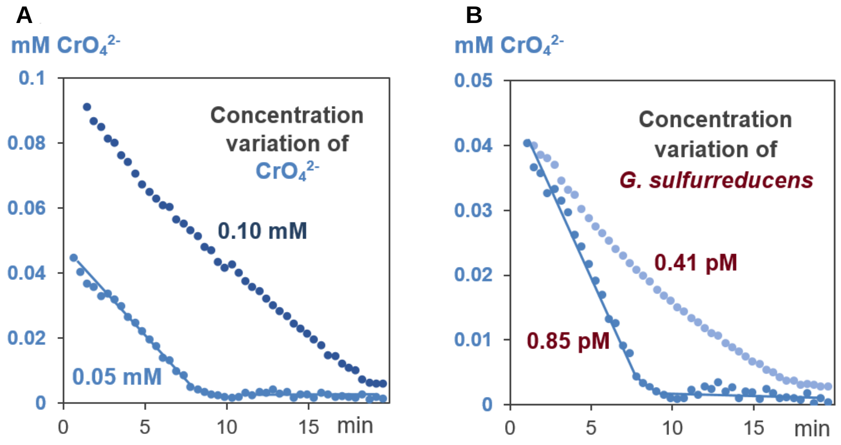

(**A**) Respiration times increased when CrO_4^{2-} concentrations were increased. (**B**) Respiration times increased when *G. sulfurreducens* concentrations were decreased. In both cases respiration rates per cell remained constant.

Fig. S6. **Fe^{2+}/heme levels during growth of *G. sulfurreducens* with fumarate as intracellular oxidant in medium A.**

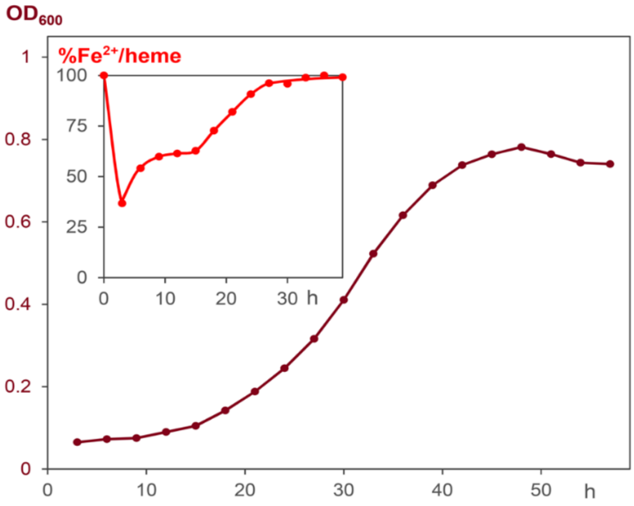

Fumarate induced growth of *G. sulfurreducens* in medium A, analyzed by UV-Vis spectroscopy. The insert shows the presence of Fe^{3+}/hemes in *c*-cytochromes during the first day of *G. sulfurreducens* growth. Oxidation experiments with CrO_4^{2-} were carried out after 30 and 36 hours of growth. At these times the iron hemes were in the Fe^{2+} oxidation state at the reaction start.
